# Direct Radiofrequency Application Improves Pain and Gait in Collagenase-Induced Acute Achilles Tendon Injury

**DOI:** 10.1155/2013/402692

**Published:** 2013-11-20

**Authors:** Yun-Pu Tsai, Chi-Wen Chang, Jung-Shun Lee, Jen-I Liang, Tsung-Hsun Hsieh, Ming-Long Yeh, Chun-I Sze

**Affiliations:** ^1^Department of Cell Biology and Anatomy, College of Medicine, National Cheng Kung University, No. 138, Sheng-Li Road, Tainan 704, Taiwan; ^2^Department of Surgery, National Cheng Kung University Affiliated Hospital, Tainan 704, Taiwan; ^3^Medical Device Innovation Center, College of Medicine, National Cheng Kung University, No. 138, Sheng-Li Road, Tainan 704, Taiwan; ^4^Department of Pathology, College of Medicine, National Cheng Kung University, No. 138, Sheng-Li Road, Tainan 704, Taiwan

## Abstract

Radiofrequency (RF) is often used as a supplementary and alternative method to alleviate pain for chronic tendinopathy. Whether or how it would work for acute tendon injury is not addressed in the literatures. Through detailed pain and gait monitoring, we hypothesized that collagenase-induce acute tendinopathy model may be able to answer these questions. Gait parameters, including time, distance, and range of motion, were recorded and analyzed using a walking track equipped with a video-based system. Expression of substance P (SP), calcitonin gene related peptide (CGRP), and galanin were used as pain markers. Beta-III tubulin and Masson trichrome staining were used as to evaluate nerve sprouting, matrix tension, and degeneration in the tendon. Of fourteen analyzed parameters, RF significantly improved stance phase, step length, preswing, and intermediary toe-spread of gait. Improved gait related to the expression of substance P, CGRP, and reduced nerve fiber sprouting and matrix tension, but not galanin. The study indicates that direct RF application may be a valuable approach to improve gait and pain in acute tendon injury. Altered gait parameters may be used as references to evaluate therapeutic outcomes of RF or other treatment plan for tendinopathy.

## 1. Introduction

Doctors Cushing and Bovie first introduced the use of radiofrequency (RF) current in medicine about 85 years ago [1]. Today, RF energy is widely used for a number of applications, including production and supplementary treatment option for peripheral and central nervous system lesions, a variety of diseases and malignant tumors [2–5]. RF is also an alternative option used for treating chronic tendinopathy to reduce pain [6–10]. Minimal-invasive RF is capable of reducing pain after microtenotomy for tendinosis [8, 9]. RF treatment for pain associated lateral elbow tendinopathy and chronic midportion Achilles tendinopathy have been shown to be an alternative option for unsuccessful nonoperative therapies [7, 10]. Little or no inflammation presents in tendinopathy imply that pain-related cytokine released by inflammatory cells may play a minor role for pain [6]. RF thermal debridement does not affect the biomechanical properties of human cadaveric patellar tendon [11]. Immobilization helps recovery of RF-induced collagenous tissue shrinkage [12]. These reports suggest that RF is often used as an alternative and supplementary therapy for late-course tendinopathy. The effectiveness of RF is mostly obtained based on experiences in treating chronic tendinopathy in human subjects. Studies of collagenase-injected animal model in Achilles tendinopathy has been shown a long-term defective healing and poor functional test outcomes, which was evaluated at least two weeks or longer after collagenases injection [13, 14]. There is limited information available for acute tendinopathy, and effects of RF on pain relief and gait improvement for acute tendinopathy are not clearly understood. Recent reports on acute Achilles tendinopathy are focused on drugs or movement effects on pain and leg stiffness control [15]. To fill this gap, we designed the study to ask whether early RF application would reverse irreversible course of collagenase-induced tendinopathy, and whether RF is justified as an alternative option to treat acute tendinopathy. Detailed time-course of pre- and post- RF pain markers expression and gait parameters were recorded and analyzed. This model may be used for evaluating gait disturbance and determining therapeutic outcomes of RF or other treatment options in tendinopathy [16].

## 2. Materials and Methods

### 2.1. Animals and Experimental Design

 Sprague-Dawley (SD) rats were obtained from the Laboratory Animal Center, National Cheng Kung University (NCKU). All experiments were carried out and followed the University Guidelines for the Care and Use of Laboratory Animals. Adult rats weighing 273 ± 49 g; at age 12 ± 2 weeks were used for the study. Forty rats (10 normal control, 10 sham control with RF, and 20 experimental (10 with RF, 10 without RF) rats) were assigned for time-course and RF assessment. Thirty-six rats were used for time-course histological studies. Achilles tendon was uncovered by 2 mm small skin incision in all animals. Rats in experimental and sham control groups were injected either with 0.3 mg collagenase I from* Clostridium histolyticum* (Sigma-Aldrich, Ca., USA) dissolved in 20 *μ*L phosphate- buffered saline (PBS) or 20 *μ*L PBS to the midportion of the left Achilles tendon [14]. Normal controls were without treatment. The day of collagenase injection was designated as day 0. Gait parameters were recorded on post-injection days 1, 3, 5, and 7, and RF was administered to the experimental and sham-control groups on post-injection day 8, using a Universal radiofrequency generator, model URF-3AP RF stimulator (Drios Technology Inc. Ontario, Canada). The stimulator was set to pulsed mode, and the tissue adjacent to the previous needle injection-site received one pulse of stimulation at 70 volt, 2 Hz, for a duration of 30 milliseconds, at 42°C for 60 seconds. Gait parameters were recorded again on days 9, 11, 13, and 15 again. Time-course schedule also applied to rats used for histological studies. Rats were either anesthetized with 10% (W/V, 400 mg/kg) or euthanized with overdose of chloral hydrate. 

### 2.2. Gait Recording and Analysis

**Figure 1:**
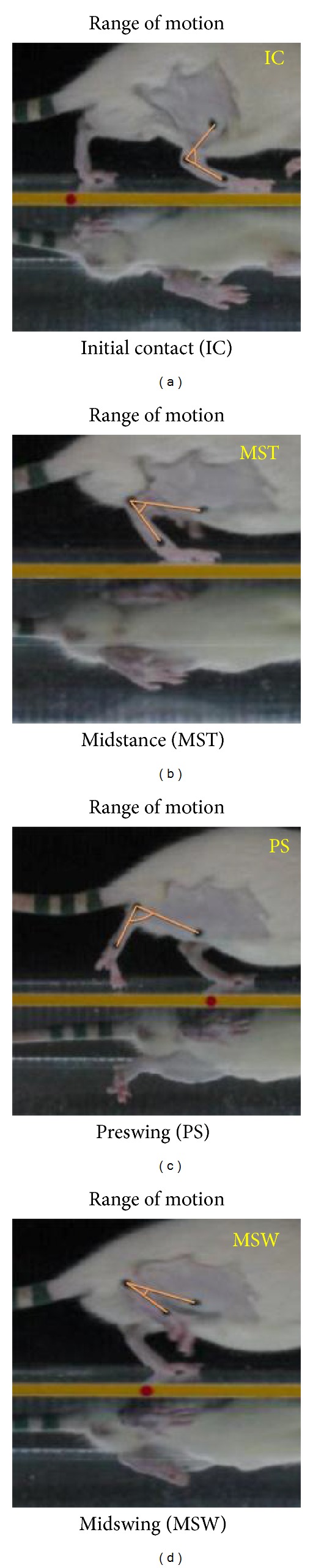
For lateral kinematical data acquisition, the hind leg of rats were shaved and marked with ink on the lateral side of skin. The marked landmarks (lateral calcaneus, fifth metatarsal head, and femoral lateral epicondyle) and angle drawn from landmarks were used for evaluating ROM.

 A walking track equipped with a video-based system was used for acquiring spatiotemporal parameters of gait [16]. The walking track apparatus consisted of a Plexiglas chamber; 80(*l*) × 6(*w*) × 12(*h*) cm with a mirror tilted at 45 degrees underneath the walking track. A digital camera (EX-F1, Casio, Japan) was coupled with the walking track to capture and record simultaneously a direct lateral view and a reflected underview of gait movements. When a rat walked to the middle 50 cm of the track, the camera was activated manually to record gait movements. The digital camera was set at 60 frames per second. For lateral kinematical data acquisition, the rats were shaved and marked with ink on the skin of the lateral side of hind limbs before each test session. The marked landmarks (lateral calcaneus, fifth metatarsal head, and femoral lateral epicondyle) and angle drawn from landmarks were used for evaluating ROM. ROM angles measurement were illustrated (Figure 1). The walking task was repeated in both directions, which allowed the recording of the movement of each hind limb. The walking task was repeated until five or six satisfactory walks of at least 4 steps without pause were obtained. Only the hind limb stepping patterns were analyzed. Three major categories of gait parameters, time, distance, and ROM were analyzed. Gait time measure is as follows: time of single foot contact the ground (stance phase), leave the ground (swing phase), both feet contact the ground (double stance phase), and walk speed. Gait distance measure is as follows: distance between opposite hind feet (step length), distance between right and left foot (step width), distance of the same hind foot when it contact and off the ground (stride length), contact length of the sole to the ground (print length), distance between 2nd and 4th toe (intermediary toe-spread), and distance between 1st and 5th toe (toe-spread). Range of motion measure ia as follows: initial angles when foot contact the ground (initial contact), standing angles of foot when opposite foot parallel to the testing foot (midstance), angles of testing foot when it leaving the ground (preswing), swing angles of testing foot when opposite foot parallel to the testing foot (midswing), and angles of sole when it contact the ground (foot angle). Tendinopathy is often associated with activity-related pain. Gait parameters vary with severity and types of tendon injury and tasks performed by the testing subjects. Abnormal gait in four legs animal could be compensate by adjust walking maneuvers. In general, weight bearing, angles of foot motions, and walking speed are major determinants to the parameters change. Achilles Functional Index (AFI) was calculated using the formula suggested by Murrell et al. [17]. 

### 2.3. Immunohistochemistry (IHC), Immunofluorescence (IF), and Masson Trichrome Staining

 Excised Achilles tendon tissues were prepared following frozen section protocols, cut at 20 *μ*m using a Shandon Cryotome E Cryostat (Thermo Scientific, German), stored at −20°C and saved for staining [18]. Tissues were stained with rabbit polyclonal SP, (Novus, USA, diluted 1 : 2000), polyclonal goat CGRP, (Santa Cruz, USA, diluted 1 : 250), polyclonal rabbit Galanin (Novus, USA, diluted 1 : 20), and DAPI (4′,6-Diamidine-2′-Phenylindole dihydrochloride) (Invitrogen, diluted 1 : 1000) antibodies. Dako LSAB plus System-HRP Kit was used to detect immunoreactivity. Monoclonal mouse Beta III tubulin antibody (Abcam, USA, diluted 1 : 1000) was used for IF staining. Alexa Fluor 488 goat antimouse (Invitrogen, diluted 1 : 200) was used for IF secondary antibody. Negative controls were treated with nonimmune IgG correspondent to the specific primary antibodies used for IHC or IF staining. Tendon tissues were stained with Masson trichrome followed the protocol recommended by the manufactures (Muto Pure Chemical Ltd., Tokyo, Japan). Microscopic images were recorded (Eclipse 80i microscope, Nikon, Japan) and saved. 

### 2.4. Image Quantification and Statistical Analysis

 GraphPad Prism software was used for two-way ANOVA and Bonferroni posttests. Gaits were analyzed by using Matlab software. Recorded gait data were normalized with control and presents as ratio to normal control. Image-pro plus 6.0 software was used to quantify tissue immunoreactivity. Five digital images with the same light exposure from worst injured areas were selected for quantitative analysis. Staining backgrounds were extracted from the images. Density, area, diameter, and ratio of intensity of density (IOD) were selected and analyzed. Gait data presented as the mean ± standard error of the mean (SEM), quantitative IHC and IF results presented as the mean ± standard deviation of the mean (SD). *P* values < 0.05 were considered statistically significant.

*P* values with asterisk represent statistical significant difference at specific day(s). *P* values without asterisk represent statistical significant of time-course studies.

## 3. Results

### 3.1. Gait Parameters in RF-Treated PBS Sham Control and Collagenase-Injected Experimental Groups

**Figure 2:**
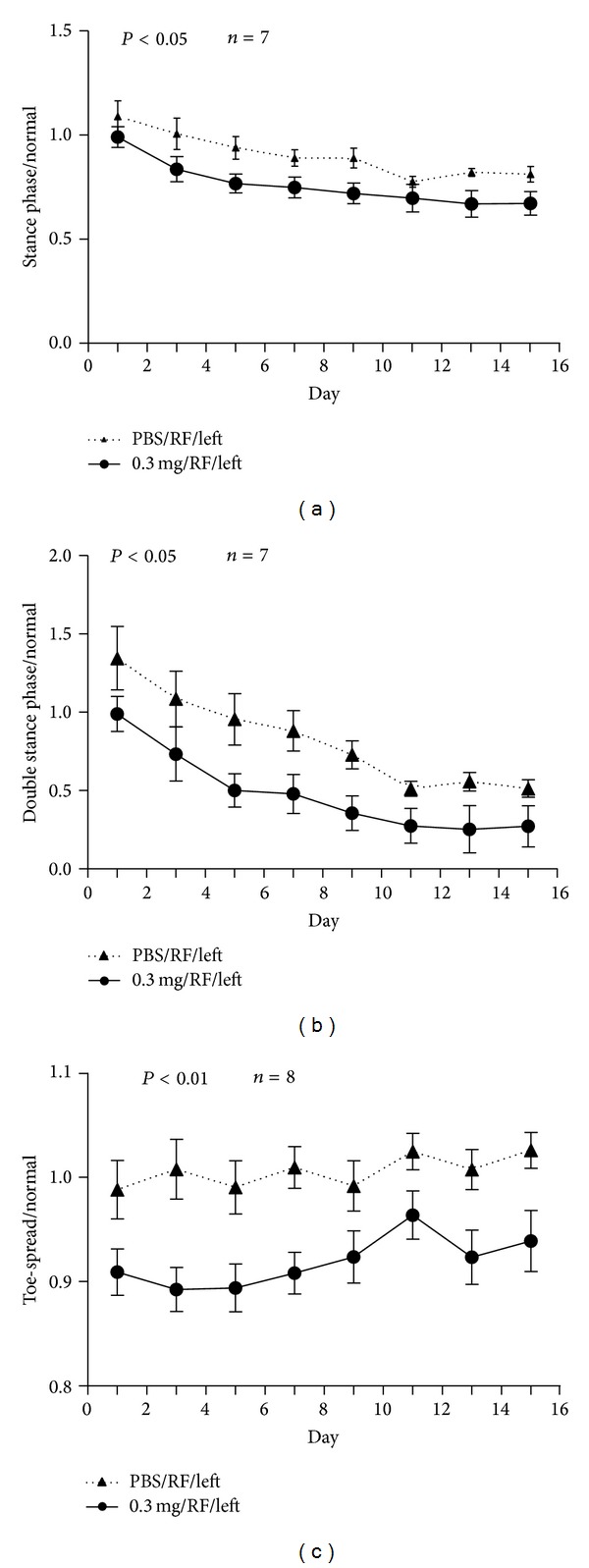
Time of stance phase and double stance were significantly decreased in experimental group when compared with PBS sham control group ((a) and (b), *n* = 7, *P* < 0.05). Toe-spread (1st–5th toe) distance decreased in RF-treated, collagenase- injected rats when compared with sham controls ((c), *n* = 8, *P* < 0.01). Data obtained from normal controls were used to normalize each gait parameter in sham and experimental groups.

Time of stance phase and double stance phases were significantly decreased in experimental group when compared with PBS sham control group (Figures 2(a) and 2(b), *n* = 7, *P* < 0.05). Toe-spread (1st–5th toe) distance was decreased in experimental group when compared with sham controls (Figure 2(c), *n* = 8, *P* < 0.01). Decrease area and injured foot contact time to the ground reducing pain. Data suggest collagenase-induced tendon injury causing more pain than tendon without collagenase injection. 

### 3.2. Gait Parameters in Collagenase-Injected Experimental Groups with or without RF

**Figure 3:**
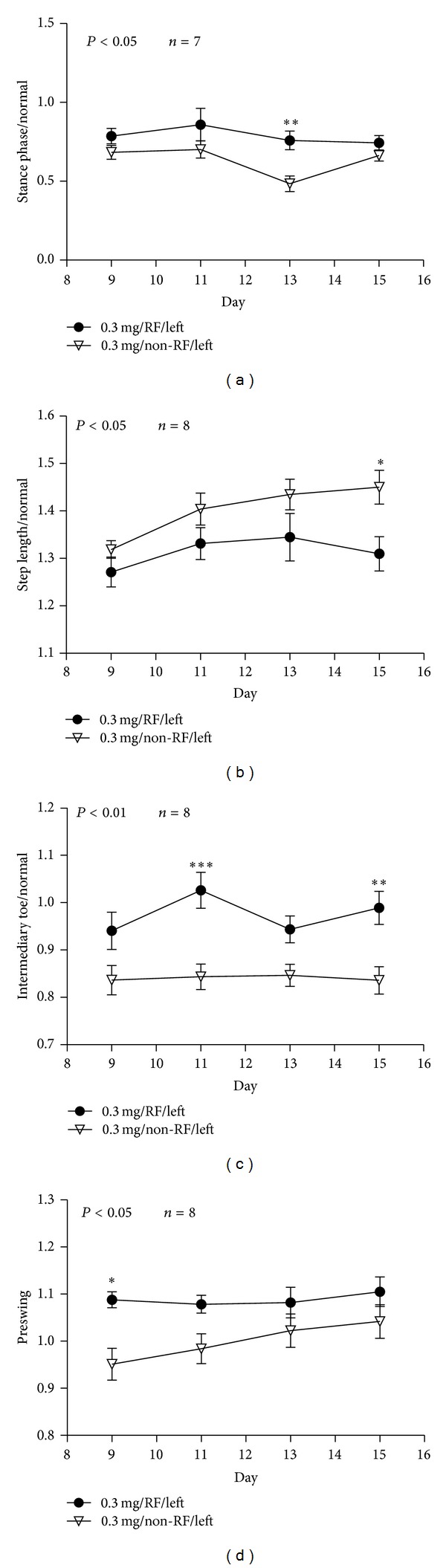
Rats of the experimental group treated with RF showed prolonged stance phase, reduced step length, and increased intermediary toe-spread distance and preswing angle to approach normal when compared with rats without RF ((a) *n* = 7, *P* < 0.05; (b) *n* = 8, *P* < 0.05; (c) *n* = 8, *P* < 0.01, and (d) *n* = 8, *P* < 0.05). When compared rats with or without RF, stance phase increased on day 13 ((a), *n* = 7, **P < 0.001); step length reduced on day 15 ((b), *n* = 8, *P < 0.05); intermediary toe spread distance increased on days 11 and 15 ((c), *n* = 8, **P < 0.001, ***P < 0.0001); preswing angle in RF group increased on days 11 and 15 ((d), *n* = 8, *P < 0.05).

RF prolonged stance phase in experimental rats when compared with rats without RF (Figure 3(a), *n* = 7, *P* < 0.05). On day 13, RF significantly prolonged time of stance phase when compared with rat without RF (Figure 3(a), *n* = 7, **P < 0.001). RF reduced step length toward to normal when compared with rats without RF (Figure 3(b), *n* = 8, *P* < 0.05). On day 15, step length was significantly reduced (Figure 3(b), *n* = 8, *P < 0.05). RF increased intermediary toe-spread (2nd–4th toe) distance toward to normal when compared with rats without RF (Figure 3(c), *n* = 8, *P* < 0.01); it was significantly increased and approach to normal on days 11 and 15 (Figure 3(c), *n* = 8, **P < 0.001, ***P < 0.0001). RF improved preswing angles toward to normal when compared rats or without RF (Figure 3(d), *n* = 8, *P* < 0.05). On day 9, preswing angles in RF group was greater than rats without RF (Figure 3(d), *n* = 8, *P < 0.05). The results suggest that RF improves pain in collagenase injected tendon which is reflected by increase foot area and contact time to the ground, and angles of injured foot leaving ground. While RF helps to improve step length toward to normal. 

### 3.3. Ankle Range of Motion and End-Point Gait Cycle Analysis

**Figure 4:**
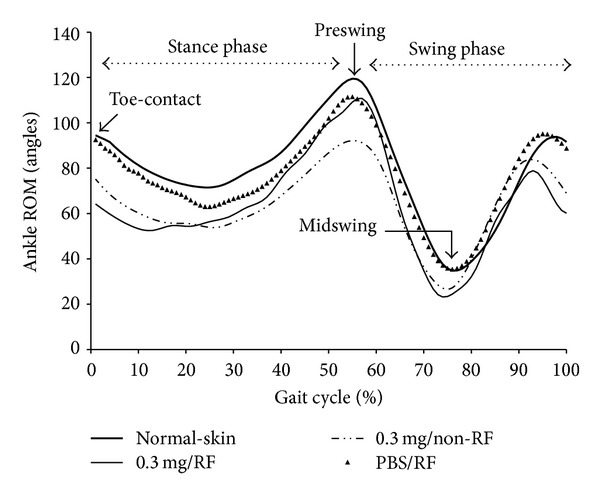
The pre- and midswing angles are reduced in the collagenase-treated group when compare to normal controls. RF treatment improves preswing angle.

The preswing angles in sham and experimental groups that underwent RF treatment were greater than non-RF groups, but smaller than normal control group (Figure 4). The midswing angles in collagenase-injected rats with or without RF were smaller than PBS sham controls with RF and normal control groups. RF treatment had no significant effect on midswing phase (Figure 4). End-point gait cycle analysis on day 15 showed RF improved step length and intermediary toe-spread (Table 1). Gait distance and area of foot contact are determinants to evaluate effectiveness of RF in acute Achilles tendinopathy. 

### 3.4. Expression of Pain Markers

**Figure 5:**
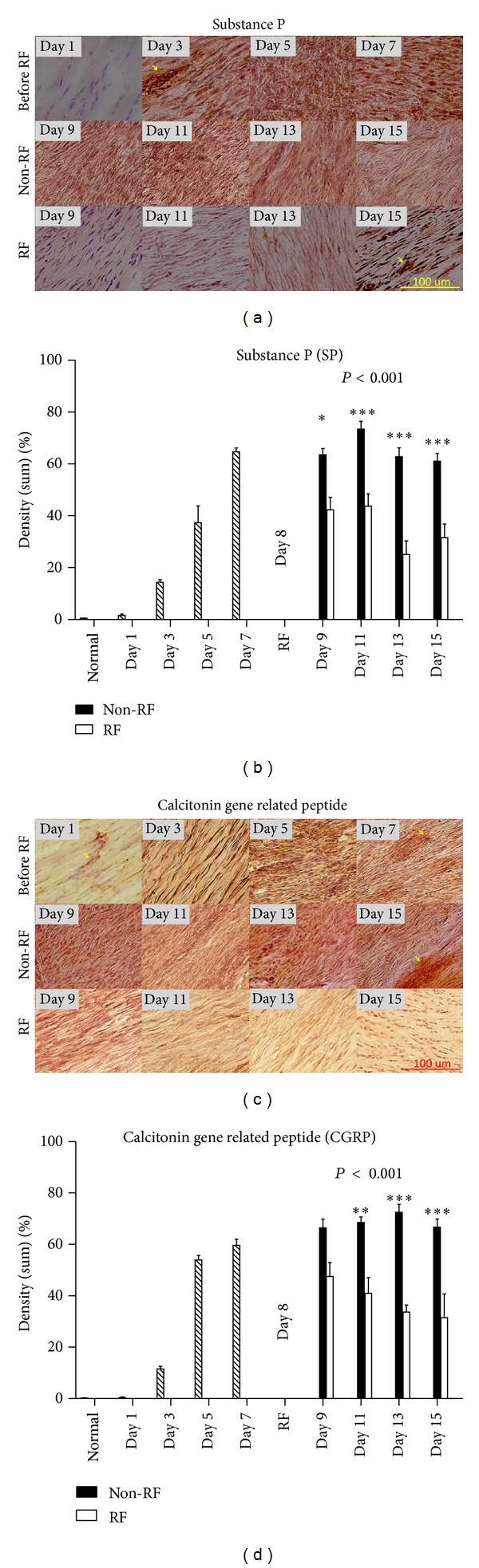
SP and CGRP stained brown in high cellularity areas. RF-treated tissues stained less SP and CGRP than tendons without RF ((a) and (b)). SP and CGRP expression were significantly reduced in RF-treated tendons ((c) and (d), *n* = 5, *P* < 0.05 and *P* < 0.001, resp.). Pain markers expression were gradually reduced; on days 9, 11, 13, and 15 for SP, and days 11, 13, and 15 for CGRP ((c) and (d), *P < 0.05, **P < 0.001, ***P < 0.0001). Blood vessels stained darker than matrix (arrows). Magnification, 200x; scale bar, 100 *μ*m.

IHC staining showed SP and CGRP stained brown in high cellularity areas. RF-treated tendons expressed less SP and CGRP than non-RF-treated tendons (Figures 5(a) and 5(b)). SP and CGRP expression in RF-treated tendons were significantly less than non-RF-treated tendons (Figures 5(c) and 5(d), *n* = 5, *P* < 0.05 and *P* < 0.001, resp.). Post-RF pain markers expression were gradually reduced; on days 9, 11, 13, and 15 for SP, and days 11, 13, and 15 for CGRP (Figures 5(c) and 5(d), *P < 0.05, **P < 0.001, ***P < 0.0001). RF reduces pain which is reflected by the levels of pain markers expression on specific days.

### 3.5. Alteration of Nerve Fiber and Achilles Tendon Matrix

**Figure 6:**
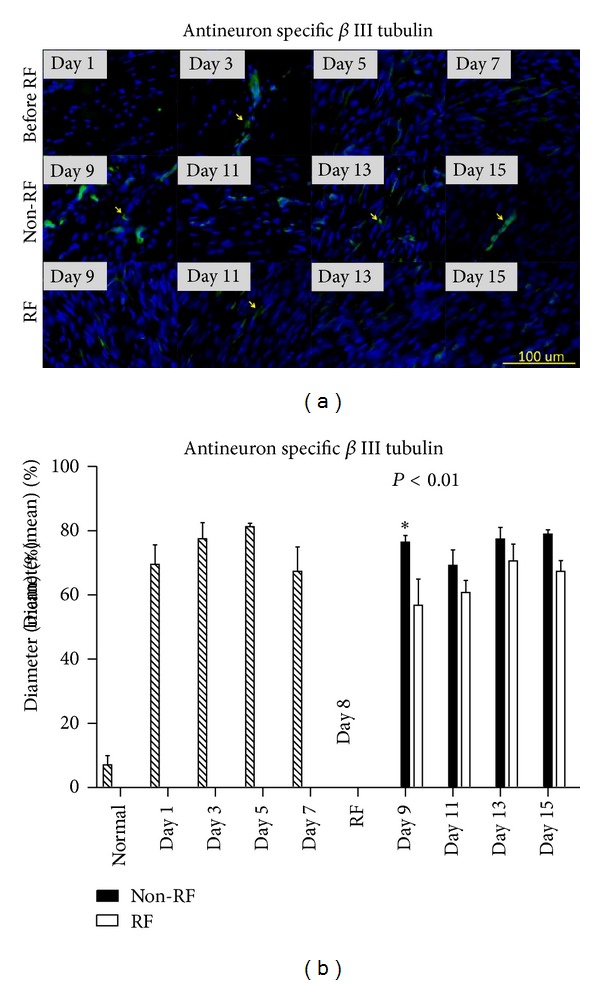
Nerve fibers stained green (arrows) and fibroblast nuclei stained blue in tendon matrix. Nerve fibers sprouting occurred mainly in high cellularity areas that have fibroblasts proliferation. Lesser nerve fibers were observed in RF-treated tendons (a). RF significantly reduced nerve fibers diameter ((b), *n* = 5, *P* < 0.01). On day 9, nerve fiber diameter was significantly decreased ((b), *n* = 5, *P < 0.05). With or without RF nerve fiber diameter has a tendency to increase after day 9 ((b)). Magnification, 200x; scale bar, 100 *μ*m.

**Figure 7:**
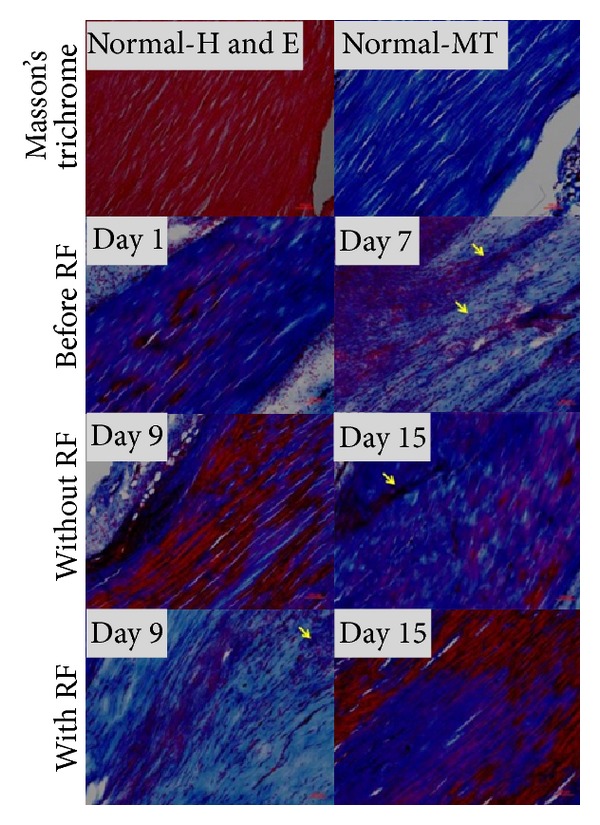
Masson trichrome (MT) showed increased fibroblasts nuclei (arrows) and collagen which stained black and blue in high cellularity areas (day 1 and 7). On day 9, areas of red stained matrix were reduced in RF-treated tendons when compared with tendon without RF. On day 15, red stained matrix reappeared in tendons with or without RF. Magnification, 100x; scale bar, 100 *μ*m.

Beta III tubulin IF staining showed nerve fibers stained green in tendon matrix. Oval to elliptical shaped fibroblast nuclei stained blue. Pain markers expression and nerve fibers sprouting occurred mainly in areas of fibroblasts proliferation. RF-treated tendon showed lesser nerve fibers when compared with collagenase-injected tendon without RF (Figure 6(a)). RF significantly reduced nerve fibers diameter; however, it had tendency to recovery (Figure 6(b), *n* = 5, *P* < 0.01). On time-course study day 9, nerve fiber diameter was significantly decreased (Figure 6(b), *n* = 5, *P < 0.05). RF treatment had no significant effect on nerve fiber density. In collagenase-injected tendon, Masson trichrome staining showed progressively increased fibroblasts and collagen which stained black and blue in high cellularity areas (Figure 7, day 1 and 7). On time-course study day 9, red stained matrix areas were reduced when compared with tendons with or without RF (Figure 7, day 9). On time-course study day 15, red stained matrix reappeared in tendons with or without RF (Figure 7, day 15). RF reduces aberrant small nerve fibers sprouting. Masson trichrome reveal RF improves tendon tension in certain days. Reappearing red matrix staining indicates that collagenase injection may cause continuous injury to the tendon. 

## 4. Discussion 

RF is one of alternative methods to alleviate pain for chronic tendinopathy. Tendinopathy without appropriate management may complicate by irreversible damage to the tendon matrix, and requires surgery [19]. It is clinical relevant to understand how RF works, why post-RF patient develop relapse pain, and which gait parameter maybe used for evaluating RF outcomes. 

Antalgic gait maneuvers are used by patients or animals with knee or foot injury in a spatiotemporal manner, which is often accompanied with subnormal knee motion during stance and swing phases [20–22]. In order to complete a walking task, antalgic maneuvers reduce the amount of time an injured foot contacts to the ground. Gait stance and double stance phases were shortened when comparing experimental with sham control groups, both with RF treatment (Figures 2(a) and 2(b)). However, stance phase was prolonged in collagenase-injected and RF-treated rats when compared with rats without RF (Figure 3(a)). Stance phase bears the weight of the body, and accounts for about 50% of a gait cycle during which the foot is on the ground (Figure 4). Gait-stance is one of the valid methods to measure severity and recovery of peripheral injury [22]. Double stance phase is a sensitive parameter to characterize pain [23, 24]. Our data demonstrate that by shortening contact time of gait stance and double stance phase to the ground on the injured side may alleviate pain. It also suggests that collagenase injection causes more pain than sham controls. RF improves pain in tendon injected with collagenase than tendon without RF. These findings suggest RF alleviates pain to allow injured limbs to spend more time in contact with the ground while walking. Results of histological studies support that decreased of pain markers expression, recovery of matrix tension, and reduction of abnormal nerve sprouting all may contribute to the improvement of gait and pain (Figures 5, 6 and 7) [25–27]. 

 In normal walking, the length of a footstep is equal on both sides. When one foot is injured, animal develops a compensating antalgic gait maneuver. Typically, step length increases on the injured foot while step length decreases on the non-injured side [20]. Post-RF reduces step length toward normal ratio or close to the noninjured site is consistent with previous observations (Figure 3(b)). On the contrary, our data showed that RF increases toe-spread (2nd–4th toe) distance and preswing angle (Figures 3(c) and 3(d)). Altered toe-spread distance changes torque and area of a foot that support body weight [28]. Swing phase defines the period of time when the foot is not in contact with the ground. Preswing phase begins at the termination of stance phase. Preswing phase is a critical period of a gait for muscles to accomplish subtasks of walking, which includes improving walking speed, swing initiation, and power generation [29–31]. RF treatment increases preswing angle and intermediary toe-spread distance approaching normal, which may improve swing initiation, muscle power generation, gait, and reducing pain. End-point gait cycle analysis on day 15 showed that RF treatment improves step length and intermediary spread but have no significant effects on preswing angle (Table 1). It reflects continuous changes in gait parameters, matrix tension, and expression of pain markers. Relapse of pain and abnormal gait parameters are coping with fluctuate pain markers expression and continue nerve fibers diameter changes (Figures 5–7). 

 Collagenase injection results in tendinocytes destruction, fibroblast proliferation, increased nerve fiber sprouting and matrix tension, all of which may impair tendon spring function [32, 33]. These pathological changes cause pain, hinder muscle contraction, and interfere with gait. Our data demonstrate that RF alone cannot completely eradicate nerve fiber sprouting, stop fibroblast proliferation, and reverse tension in matrix (Figures 5–7). It explains the occurrence of relapsed pain and irreversible damage in tendinopathy model [32, 33]. Chondrocyte-like cells or tissue calcification which is indicative of irreversible damage is not identified in our acute rat model [19]. However, it occurs 4 weeks after collagenase injection (data not shown). Wether repeat RF applications, RF combines with other options, or design therapeutic regimes targets acute fibrous degeneration/regeneration and nerve sprout would enhance RF efficacy requires further investigation.

## 5. Conclusion

Time-course gait and pain markers studies demonstrate a continuous and dynamic nature of acute tendinopathy. Body weight loading, time, and areas of foot contact to the ground of injured foot are key parameters to determine how animal walks and responds to pain. The pathophysiology contents of injured tendon determine the final outcome of RF treatment. AFI is not impaired in our model suggesting that gait can be compensated by noninjured feet. Our model provides dynamic analysis of compensatory antalgic gait mechanisms in animal that may be employed to patients suffering from foot pain. Given continuous histopathological changes in the matrix, our model may mimic acute and sever Achilles tendon injury when too much force cause partially or completely tear or rupture to the tendon. This model may not fully represent tendinopathy; however, it has advantage of quickly and conveniently inducing abnormal gait and pathohistological changes observed in tendinopathy. Gait step length and intermediary toe-spread may be candidate parameters to evaluate RF outcomes in acute Achilles tendon injury. Finally, direct RF application works for acute tendon injury to improve gain and alleviate pain. 

## Figures and Tables

**Table 1:** Results of end-point gait cycle analysis on day 15.

	Normal	Non-RF	RF	Normal versus Non-RF	Normal versus RF	Non-RF versus RF
Stance phase (ms)	235.15 ± 36.26	191.83 ± 29.83	194.44 ± 49.08	NS	NS	NS
Step length (mm)	83.9327 ± 6.66	95.60 ± 6.63	86.23 ± 7.11	**	NS	*
Intermediary toe-spread (mm)	9.81 ± 1.41	8.32 ± 0.90	9.84 ± 0.98	*	NS	*
Preswing (°)	113.39 ± 8.13	99.60 ± 10.73	105.65 ± 8.50	*	NS	NS

ms: millisecond, mm: millimeter; °range of motion angle; NS: not significant.

*P < 0.05; **P < 0.001. Data are presented as mean ± standard deviation.
